# Significant Involvement of Double Diffusion Theories on Viscoelastic Fluid Comprising Variable Thermophysical Properties

**DOI:** 10.3390/mi12080951

**Published:** 2021-08-12

**Authors:** Muhammad Sohail, Umar Nazir, Omar Bazighifan, Rami Ahmad El-Nabulsi, Mahmoud M. Selim, Hussam Alrabaiah, Phatiphat Thounthong

**Affiliations:** 1 Department of Applied Mathematics and Statistics, Institute of Space Technology, P.O. Box 2750, Islamabad 44000, Pakistan; nazir_u2563@yahoo.com; 2 Section of Mathematics, International Telematic University Uninettuno, CorsoVittorio Emanuele II, 39, 00186 Roma, Italy; o.bazighifan@gmail.com; 3 Research Center for Quantum Technology, Faculty of Science, Chiang Mai University, Chiang Mai 50200, Thailand; 4 Department of Physics and Materials Science, Faculty of Science, Chiang Mai University, Chiang Mai 50200, Thailand; 5 Athens Institute for Education and Research, Mathematics and Physics Divisions, 8 Valaoritou Street, Kolonaki, 10671 Athens, Greece; 6 Department of Mathematics, Al-Aflaj College of Science and Humanities Studies, Prince Sattam Bin Abdulaziz University, Al-Aflaj 710-11912, Saudi Arabia; m.selim@psau.edu.sa; 7 Department of Mathematics, Suez Faculty of Science, Suez University, Suez 34891, Egypt; 8 College of Engineering, Al Ain University, Al Ain P.O. Box 15551, United Arab Emirates; hussam.alrabaiah@aau.ac.ae; 9 Department of Mathematics, Tafila Technical University, Tafila 66110, Jordan; 10 Renewable Energy Research Centre, Department of Teacher Training in Electrical Engineering, Faculty of Technical Education, King Mongkut’s University of Technology North Bangkok, 1518 Pracharat 1 Road, Bang Sue, Bangkok 10800, Thailand; phtt@kmutnb.ac.th

**Keywords:** viscoelastic material, group similarity analysis, thermal relaxation time, parametric investigation, variable magnetic field, error analysis

## Abstract

This report examines the heat and mass transfer in three-dimensional second grade non-Newtonian fluid in the presence of a variable magnetic field. Heat transfer is presented with the involvement of thermal relaxation time and variable thermal conductivity. The generalized theory for mass flux with variable mass diffusion coefficient is considered in the transport of species. The conservation laws are modeled in simplified form via boundary layer theory which results as a system of coupled non-linear partial differential equations. Group similarity analysis is engaged for the conversion of derived conservation laws in the form of highly non-linear ordinary differential equations. The solution is obtained vial optimal homotopy procedure (OHP). The convergence of the scheme is shown through error analysis. The obtained solution is displayed through graphs and tables for different influential parameters.

## 1. Introduction

Fluid flows over stretched surfaces have applications in several fields and has significant involvement in the practical usage of several items. Scientists and engineers have made efforts to explore their features and usage in different processes. The mathematical relations of non-Newtonian materials are different as compared with Newtonian material. These materials are divided into different categories according to their properties. An important non-Newtonian fluid is a second grade fluid [1,2,3,4,5,6,7]. It has the following constitutive relation:

$$\tau^{*}=-PI+\mu F_{1}+\beta_{1}F_{2}+\beta_{2}F_{1}*F_{1},\;\beta_{1}\geq 0,\;\mu \geq 0,\;\beta_{1}+\beta_{2}=0.$$

Hayat et al. [1] studied the mixed convection in the second grade model over a stretching cylinder. They modeled the problem in two dimensions with thermal transport by taking the variable thermal conductivity. They used a homotopy method for the solution. They studied the contribution of several emerging parameters on the solution through graphs. They noticed the decrease in velocity field for a mixed convection parameter. Massoudi et al. [2] reported the study on the second grade model with temperature dependent viscosity between parallel plates. They presented a comparative study for the validity of obtained solution through tabular data. An exact solution through an oscillating sphere for a second grade model was computed by Fetecau et al. [3]. They found that the solution is periodic and it is independent of initial data. Hankel and Laplace transforms were engaged by Kamran et al. [4] to handle the modeled equations for fractional second grade model in cylindrical coordinates. They presented that the fractional model present the fluid flow phenomenon more accurately as compared with the ordinary derivatives. Chauhan and Kumar [5] studied the unsteady mechanics for second grade model in partially filled porous channel. They used a Laplace transform technique to analyze the solution. They observed the increase in velocity field against time parameter. A rotating viscoelastic model with ramped wall temperature condition for exact solution was reported by Mohamad et al. [6]. They plotted the solution against numerous emerging parameters. They noticed the dual behavior of velocity against the rotation parameter. Hayat et al. [7] examined the comportment of chemical reaction with solutal and thermal transport in a second grade model passed over a bi-directional stretched surface. They found the increase in dimensionless stress against ratio parameter and viscoelastic parameter. Moon et al. [8] discussed the phenomenon of heat transfer and Weber number including droplets of xanthan gum solution (non-Newtonian) and DI-water (Newtonian) over a heated surface. They noted that the DI-water droplet has higher spreading diameter as compared with the non-Newtonian (droplet) because of variation in fluid difference. German and Bertola [9] studied free-fall related to the liquid drops due gravity of force. They imagined high speed of drops based on viscoelastic fluids. They found that shape of the drop is changed under the action of yield stress. An and Lee [10] experimentally discussed the oscillations (free falling) of drops (shear-thinning) due to the force of gravity based on viscoelastic fluids passed over a solid surface. Moon et al. [11] suggested a mixed regime (coalescence occurs) using sequential images and mixed regime is exaggerated due to volumes of static droplet volumes, static droplets and Weber numbers. They estimated the film thickness (between two drops) via lubrication theory. Zhao and Khayat [12] discussed the flow behavior in view of shear-thickening and shear-thinning of a jet (non-Newtonian) over a flat plate via a hydraulic jump. Moon et al. [13] considered the features of droplets (non-Newtonian) on solid surfaces considering various Weber numbers. They considered xanthan gum solution to produce the droplets (non-Newtonian) measured via spreading diameters and camera (high speed).

Transportation of heat has applications in different engineering aspects and it is now a hot topic for researchers working in the field of engineering and applied mathematics. Several researchers are working on transport phenomena actively. For instance, Naseem et al. [14] worked on third grade nanofluid passed over a Riga plate. They considered several physical effects while modelling the transport equations and the resulting equations were simplified via boundary layer theory with the solution approximated analytically. They noticed the rise in velocity profile for modified magnetic parameter and Reynolds number. Sahoo and Poncet [15] studied the Blasius flow of a fourth grade model with heat transfer in a porous permeable stretching surface. Heat transfer in MHD viscous stagnation point dusty fluid with a non-uniform heat source in a porous stretching sheet was studied by Ramesh et al. [16]. They presented a comparative analysis and solved the resulting equations with the help of a shooting method. They discussed the contribution of several parameters on velocity and temperature fields. They noticed the decline in thermal field against higher Prandtl number. Qiu et al. [17] studied the thermal transport in channel via finite volume technique. They also reported the analysis of entropy in their findings. They discussed the impact of nanoparticles volume fraction on flow and entropy. Khan et al. [18] studied the heat transfer in a stagnation point Powell–Eyring model in a stretching cylinder with variable properties analytically. They observed the enhancement in temperature and velocity fields against the growing values or curvature parameter. Also they listed the numerical values for heat transfer coefficient against different parameters. Few recent studies covering non-linear transport problems with different effects have been reported in [19,20,21,22,23,24,25].

The objective of current inspection is to analyze the comportment of variable properties in heat and mass transportation in a second grade steady incompressible model past over a bi-directional elongating surface. This article is organized as follows: Section 1 contains the literature survey; modeling of considered problem is included in Section 2 with important physical quantities; Section 3 comprises methodology and results with key findings covered in Section 4 and Section 5 respectively.

## 2. Mathematical Drafting of Viscoelastic Fluid with Thermal and Mass Transport

**Figure 1:**
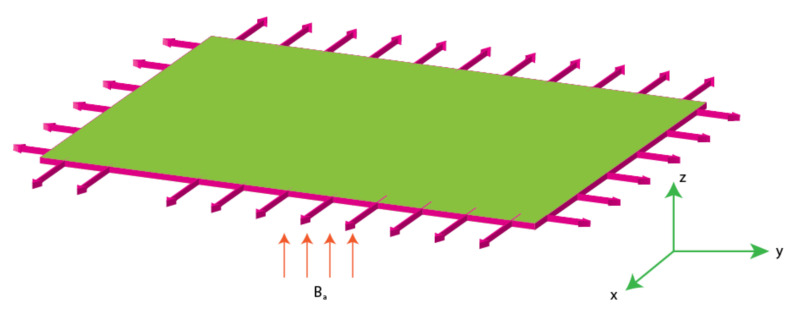
Geometry of second grade fluid model.

An analysis of the transport phenomenon on a second grade fluid [7,14] over a bi-directional elastic surface is presented in Figure 1. It is assumed that the sheet is stretched along x- and y- directions, respectively, and flow occupies the region normal to *x*- and *y*-axis. The sheet is kept at temperature “$T_{w}$” and concentration “$C_{w}$”. Along *x*-axis, the velocity is $“U_{W}=ax”$ and $“V_{W}=by”$ is along the y-axis. The following important considerations have been adopted to derive the conservation laws

❖ Three-dimensional flow;
❖ Bi-directional elastic surface;
❖ Incompressible fluid;
❖ Steady flow;
❖ Viscoelastic second grade fluid;
❖ Heat flux via generalized theory of Cattaneo–Christov;
❖ Temperature-dependent thermal conductivity model;
❖ Space-dependent magnetic field;
❖ Updated mass flux model with temperature dependent diffusion coefficient;

The flowing resulting equations [7] appears by using the above stated assumptions

(1) $$u_{x}+v_{y}+w_{z}=0,$$

(2) $$\begin{array}{ll} uu_{x}+v\frac{\partial u}{\partial y}+w & \frac{\partial u}{\partial z}-\vartheta \frac{\partial^{2}u}{\partial z^{2}}\\ & +\alpha_{0}[u\frac{\partial^{3}u}{\partial x\partial z^{2}}+w\frac{\partial^{3}u}{\partial z^{3}}-\frac{\partial u}{\partial x}\frac{\partial^{2}u}{\partial z^{2}}-\frac{\partial u}{\partial z}\frac{\partial^{2}w}{\partial z^{2}}-2\frac{\partial u}{\partial z}\frac{\partial^{2}u}{\partial x\partial z}\\ & -2\frac{\partial w}{\partial z}\frac{\partial^{2}u}{\partial z^{2}}]+\frac{\sigma }{\rho }B_{a}^{2}\left(x,y\right)u=0, \end{array}$$

(3) $$\begin{array}{ll} uv_{x}+v\frac{\partial v}{\partial y}+w & \frac{\partial v}{\partial z}-\vartheta \frac{\partial^{2}v}{\partial z^{2}}\\ & +\alpha_{0}[v\frac{\partial^{3}v}{\partial x\partial z^{2}}+w\frac{\partial^{3}v}{\partial z^{3}}-\frac{\partial v}{\partial x}\frac{\partial^{2}v}{\partial z^{2}}-\frac{\partial v}{\partial z}\frac{\partial^{2}w}{\partial z^{2}}-2\frac{\partial v}{\partial z}\frac{\partial^{2}v}{\partial x\partial z}\\ & -2\frac{\partial w}{\partial z}\frac{\partial^{2}v}{\partial z^{2}}]+\frac{\;\sigma }{\rho }B_{a}^{2}\left(x,y\right)v=0, \end{array}$$

(4) $$uT_{x}+vT_{y}+wT_{z}+\alpha_{a}\left[\begin{array}{c} \left(uu_{x}+vu_{y}+wu_{z}\right)T_{x}+\left(uv_{x}+v\frac{\partial v}{\partial y}+w\frac{\partial v}{\partial z}\right)T_{y}\\ +\left(uw_{x}+v\frac{\partial w}{\partial y}+w\frac{\partial w}{\partial z}\right)T_{z}+2uvT_{xy}+2vwT_{yz}\\ +2uwT_{xz}+u^{2}T_{xx}+v^{2}T_{yy}+w^{2}T_{zz} \end{array}\right]\;-\nabla \left[K_{A}\left(T\right)\nabla T\right]=0\;$$

(5) $$uC_{x}+vC_{y}+wC_{z}+\alpha_{b}\left[\begin{array}{c} \left(uu_{x}+v\frac{\partial u}{\partial y}+w\frac{\partial u}{\partial z}\right)C_{x}+\left(uv_{x}+v\frac{\partial v}{\partial y}+w\frac{\partial v}{\partial z}\right)C_{y}\\ +\left(uw_{x}+v\frac{\partial w}{\partial y}+w\frac{\partial w}{\partial z}\right)C_{z}+2uvC_{xy}+2vwC_{yz}\\ +2uwC_{xz}+u^{2}C_{xx}+v^{2}C_{yy}+w^{2}C_{zz} \end{array}\right]\;-\nabla \left[D_{A}\left(T\right)\nabla C\right]=0.\;$$

Boundary conditions for the dimensional problem are

(6) $$\left\{\begin{array}{l} u=U_{W}=ax,\;v=V_{W}=by,w=0,\;T=T_{w},\;C=C_{w}\;\mathrm{at}\;z=0.\\ u\to 0,\;v\to 0,\;T\to T_{\infty },\;C\to C_{\infty }\;for\;z\to \infty . \end{array}\right.$$

With the use of the following similarity variables, the governing law reduce to

(7) $$\left\{\begin{array}{c} u=axf^{\prime }\left[\eta \right],\;v=ayg^{\prime }\left[\eta \right],w=-{\left(a\vartheta \right)}^{\frac{1}{2}}\left[f\left[\eta \right]+g\left[\eta \right]\right],\;\\ \theta \left[\eta \right]=\frac{T-T_{\infty }}{T_{w}-T_{\infty }},\;\phi \left[\eta \right]=\frac{C-C_{\infty }}{C_{w}-C_{\infty }},\;\left[\eta \right]={\left(\frac{a}{\vartheta }\right)}^{\frac{1}{2}}z, \end{array}\right.$$

(8) $$-Mf^{\prime }\left[\eta \right]-f^{\prime }{\left[\eta \right]}^{2}+\left(f\left[\eta \right]+g\left[\eta \right]\right)f^{\prime \prime }\left[\eta \right]+f^{\left(3\right)}\left[\eta \right]+R\left(f^{\prime \prime }\left[\eta \right]\left(f^{\prime \prime }\left[\eta \right]-g^{\prime \prime }\left[\eta \right]\right)\;\;\;\;\;-2\left(f^{\prime }\left[\eta \right]+g^{\prime }\left[\eta \right]\right)f^{\left(3\right)}\left[\eta \right]+\left(f\left[\eta \right]+g\left[\eta \right]\right)f^{\left(4\right)}\left[\eta \right]\right)=0,$$

(9) $$-Mg^{\prime }\left[\eta \right]-g^{\prime }{\left[\eta \right]}^{2}+\left(f\left[\eta \right]+g\left[\eta \right]\right)g^{\prime \prime }\left[\eta \right]+g^{\left(3\right)}\left[\eta \right]+R\left(\left(f^{\prime \prime }\left[\eta \right]-g^{\prime \prime }\left[\eta \right]\right)g^{\prime \prime }\left[\eta \right]\;\;\;\;\;-2\left(f^{\prime }\left[\eta \right]+g^{\prime }\left[\eta \right]\right)g^{\left(3\right)}\left[\eta \right]+\left(f\left[\eta \right]+g\left[\eta \right]\right)g^{\left(4\right)}\left[\eta \right]\right)=0,$$

(10) $$\left(f\left[\eta \right]+g\left[\eta \right]\right)\theta^{\prime }\left[\eta \right]+\frac{1}{Pr}\left(1+\gamma_{1}\theta \left[\eta \right]\right)\theta^{\prime \prime }\left[\eta \right]-\alpha_{1}\left(f\left[\eta \right]+g\left[\eta \right]\right)\left(f^{\prime }\left[\eta \right]\;\;\;\;\;+g^{\prime }\left[\eta \right]\theta^{\prime }\left[\eta \right]+\left(f\left[\eta \right]+g\left[\eta \right]\right)\theta^{\prime \prime }\left[\eta \right]\right)=0,$$

(11) $$\left(f\left[\eta \right]+g\left[\eta \right]\right)\phi^{\prime }\left[\eta \right]+\frac{1}{Sc}\left(1+\gamma_{2}\theta \left[\eta \right]\right)\phi^{\prime \prime }\left[\eta \right]-\alpha_{2}\left(f\left[\eta \right]+g\left[\eta \right]\right)\left(f^{\prime }\left[\eta \right]\;\;\;\;\;+g^{\prime }\left[\eta \right]\phi^{\prime }\left[\eta \right]+\left(f\left[\eta \right]+g\left[\eta \right]\right)\phi^{\prime \prime }\left[\eta \right]\right)=0,$$

(12) $$\left\{\begin{array}{l} f\left(0\right)=0,\;g\left(0\right)=0,\;f^{\prime }\left(0\right)=1,\;g^{\prime }\left(0\right)=\delta ,\;\theta \left(0\right)=1,\;\phi \left(0\right)=1,\\ f^{\prime }\left(\infty \right)=0,\;g^{\prime }\left(\infty \right)=0,\;\theta \left(\infty \right)=0,\;\phi \left(\infty \right)=0. \end{array}\right.$$

### Physical Quantities

The study of heat, mass transfer rates and dimensionless stress at the boundary has significant applications and usage in industry. Therefore, scientists and engineers are keenly observing their features against different physical parameters which influence them directly. These quantities are defined as:

(13) $$C_{XF}=\frac{{\left.\tau_{xz}\right|}_{z}=0}{\rho {\left(u_{w}\right)}^{2}},C_{YF}=\frac{{\left.\tau_{yz}\right|}_{z}=0\;}{\rho {\left(v_{w}\right)}^{2}},$$

(14) $$\tau_{xz}={\left[\mu \frac{\partial u}{\partial z}+\alpha_{0}\left[u\frac{\partial^{2}u}{\partial x\partial z}+v\frac{\partial^{2}u}{\partial y\partial z}+w\frac{\partial^{2}u}{\partial z^{2}}+\frac{\partial u}{\partial x}\frac{\partial u}{\partial z}+\frac{\partial v}{\partial z}\frac{\partial v}{\partial x}-\frac{\partial w}{\partial z}\frac{\partial u}{\partial y}\right]\right]}_{z=0}$$

(15) $$\tau_{yz}={\left[\mu \frac{\partial v}{\partial z}+\alpha_{0}\left[u\frac{\partial^{2}v}{\partial x\partial z}+v\frac{\partial^{2}v}{\partial y\partial z}+w\frac{\partial^{2}v}{\partial z^{2}}+\frac{\partial u}{\partial y}\frac{\partial u}{\partial z}+\frac{\partial v}{\partial z}\frac{\partial v}{\partial y}-\frac{\partial w}{\partial z}\frac{\partial v}{\partial y}\right]\right]}_{z=0},$$

(16) $$Nu_{xy}=\frac{\left(x+y\right)Q_{w}^{*}}{K\left(T\right)\left[T_{w}-T_{\infty }\right]},\;Q_{w}^{*}=-K\left(T\right)\frac{\partial T}{\partial z}{|}_{z=0},$$

(17) $$Su_{xy}=\frac{\left(x+y\right)M_{w}^{*}}{D_{B}\left(T\right)\left[C_{w}-C_{\infty }\right]},\;M_{w}^{*}=-D_{B}\left(T\right)\frac{\partial C}{\partial z}{|}_{z=0}$$

After boundary layer theory, the dimensionless form is:

(18) $$C_{Fx}^{*}=f^{\prime \prime }\left(0\right)-R\left[f\left(0\right)+g\left(0\right)\right]f^{\prime \prime \prime }\left(0\right)+R\left[f^{\prime }\left(0\right)+g^{\prime }\left(0\right)\right]\;f^{\prime }\left(0\right)\;+2Rf^{\prime }\left(0\right)f^{\prime \prime }\left(0\right),\;$$

(19) $$C_{Fy}^{*}=g^{\prime \prime }\left(0\right)-R\left[f\left(0\right)+g\left(0\right)\right]g^{\prime \prime \prime }\left(0\right)+R\left[f^{\prime }\left(0\right)+g^{\prime }\left(0\right)\right]\;g^{\prime \prime }\left(0\right)\;+2Rf^{\prime }\left(0\right)g^{\prime }\left(0\right)\;$$

(20) $$H_{xy}^{*}=-{\left(R_{e_{xy}}\right)}^{\frac{1}{2}}\theta^{\prime }\left(0\right),\;M_{xy}^{*}=-{\left(R_{e_{xy}}\right)}^{\frac{1}{2}}\phi^{\prime }\left(0\right).$$

## 3. Numerical Method for Solution

Modelling of the fluid flow problems results in the form of a set of coupled non-linear differential equations. The derived problem is highly non-linear and coupled. Due to high non-linearity, an exact solution is not possible. Researchers proposed several schemes to handle the non-linear complex differential equations. Here, optimal homotopy analysis procedure (OHAP) [7,14,19,20,21,22,23] is engaged due to its several advantages.

This section covers the necessary steps for the adopted procedure. It has the following steps:

❖ Linear operator selection;
❖ Using the boundary data;
❖ Determination of unknown constants;
❖ Adopting of initial guesses;

The linear operators with initial guesses are:

(21) $$\left\{\begin{array}{c} L_{f}^{*}=\frac{D^{3}}{D\eta^{3}}-\frac{D}{D\eta },\;L_{g}^{*}=\frac{D^{3}}{D\eta^{3}}-\frac{D}{D\eta },\;\\ L_{t}^{*}=\frac{D^{2}}{D\eta^{2}}-1,\;L_{c}^{*}=\frac{D^{2}}{D\eta^{2}}-1,\; \end{array}\right.$$

(22) $$\left\{\begin{array}{l} f_{q}\left(\eta \right)=1-e^{-\eta },\;g_{q}=\gamma \left[1-e^{-\eta }\right],\;\\ \theta_{q}\left(\eta \right)=e^{-\eta },\;\phi_{q}\left(\eta \right)=e^{-\eta }, \end{array}\right.$$

The operators in Equation (21) obeys:

(23) $$L_{f}^{*}\left[Q_{1}+Q_{2}e^{-\eta }+Q_{3}e^{\eta }\right]=0,\;L_{g}^{*}\left[Q_{4}+Q_{5}e^{-\eta }+Q_{6}e^{\eta }\right]=0,\;L_{t}^{*}\left[Q_{7}e^{-\eta }+Q_{8}e^{\eta }\right]=0,\;L_{c}^{*}\left[Q_{9}e^{\eta }+Q_{10}e^{-\eta }\right]=0.$$

where $Q_{b}\left(b=1,2,\ldots ,10\right)$ are unknowns.

Using the concepts of minimization of average squared residual error [7,8,9,10,11,12,13,14,19,20,21,22,23]:

(24) $$\delta_{m}^{f}=\frac{1}{B+1}\overset{B}{\underset{r=0}{\sum }}{\left[S_{f}\left(\overset{a}{\underset{L=0}{\sum }}\hat{f}\left(\eta \right),\;\overset{a}{\underset{L=0}{\sum }}\hat{g}\left(\eta \right)\right)\right]}^{2},$$

(25) $$\delta_{m}^{g}=\frac{1}{B+1}\overset{B}{\underset{r=0}{\sum }}{\left[S_{g}\left(\overset{a}{\underset{L=0}{\sum }}\hat{f}\left(\eta \right),\;\overset{a}{\underset{L=0}{\sum }}\hat{g}\left(\eta \right)\right)\right]}^{2},$$

(26) $$\delta_{m}^{\theta }=\frac{1}{B+1}\overset{B}{\underset{r=0}{\sum }}{\left[S_{\theta }\left(\overset{a}{\underset{L=0}{\sum }}\hat{f}\left(\eta \right),\;\overset{a}{\underset{L=0}{\sum }}\hat{g}\left(\eta \right),\;\overset{a}{\underset{L=0}{\sum }}\hat{\theta }\left(\eta \right)\right)\right]}^{2},$$

(27) $$\delta_{m}^{\phi }=\frac{1}{B+1}\overset{B}{\underset{r=0}{\sum }}{\left[S_{\phi }\left(\overset{a}{\underset{L=0}{\sum }}\hat{f}\left(\eta \right),\;\overset{a}{\underset{L=0}{\sum }}\hat{g}\left(\eta \right),\;\overset{a}{\underset{L=0}{\sum }}\hat{\theta }\left(\eta \right),\;\overset{a}{\underset{L=0}{\sum }}\hat{\phi }\left(\eta \right)\right)\right]}^{2},$$

where

(28) $$\delta_{i}^{t}=\delta_{i}^{f}+\delta_{i}^{g}+\delta_{i}^{\theta }+\delta_{i}^{\phi }.$$

The minimum error at second order is 0.00031589832079710834 and optimal values at third order are $B_{f}=-1.2160,\;B_{g}=-1.1638,\;B_{\theta }=-0.9509,\;B_{\phi }=-0.5871,$ by fixing the involved parameters as $R=0.1,Sc=0.6,\;Pr=1.1,\alpha_{1}=0.2=\alpha_{2},\;\gamma_{1}=0.3=\gamma_{2},M=0.1,\;\delta =0.8.$

(29) $$\begin{array}{ll} f=1.0-1.0e^{-z} & -0.6080M^{2}+0.6080e^{-z}M^{2}-0.6080R+0.6080e^{-z}R\\ & +0.6080e^{-z}M^{2}z+0.6080e^{-z}Rz-0.4053\delta \\ & +0.2026e^{-2z}\delta +0.2026e^{-z}\delta -1.0133R\delta \\ & -0.4053e^{-2z}R\delta +1.4187e^{-z}R\delta +0.6080e^{-z}z\delta \\ & +0.6080e^{-z}Rz\delta , \end{array}$$

(30) $$\begin{array}{ll} g=1.1939\delta & +0.1939e^{-2z}\delta -1.3879e^{-z}\delta -0.5819M^{2}\delta +0.5819e^{-z}M^{2}\delta \\ & -0.5819R\delta +0.5819e^{-z}R\delta +0.5819e^{-z}M^{2}z\delta \\ & +0.5819e^{-z}Rz\delta -0.5819\delta^{2}+0.5819e^{-z}\delta^{2}-0.9698R\delta^{2}\\ & -0.3879e^{-2z}R\delta^{2}+1.3578e^{-z}R\delta^{2}+0.5819e^{-z}z\delta^{2}\\ & +0.5819e^{-z}Rz\delta^{2}, \end{array}$$

(31) $$\begin{array}{ll} \theta =-0.3169e^{-2z} & +1.3169e^{-z}-0.4754e^{-z}z+\frac{0.47543e^{-z}z}{Pr}-0.3169e^{-2z}\delta \\ & +0.3169e^{-z}\delta -0.4754e^{-z}z\delta +0.11887e^{-3z}\alpha_{1}\\ & -0.95097e^{-2z}\alpha_{1}+0.8321e^{-z}\alpha_{1}-0.9509e^{-z}z\alpha_{1}\\ & +0.3566e^{-3z}\delta \alpha_{1}-1.9019e^{-2z}\delta \alpha_{1}+1.5453e^{-z}\delta \alpha_{1}\\ & -1.4264e^{-z}z\delta \alpha_{1}+0.2377e^{-3z}\delta^{2}\alpha_{1}-0.9509e^{-2z}\delta^{2}\alpha_{1}\\ & +0.7132e^{-z}\delta^{2}\alpha_{1}-0.475497e^{-z}z\delta^{2}\alpha_{1}-\frac{0.3169e^{-2z}\gamma_{1}}{Pr}\\ & +\frac{0.3169e^{-z}\gamma_{1},}{Pr} \end{array}$$

(32) $$\begin{array}{cc} =-0.1957e^{-2z} & +1.1957e^{-z}-0.2935e^{-z}z+\frac{0.2935e^{-z}z}{\mathrm{Sc}}-0.1957e^{-2z}\delta \\ & +0.1957e^{-z}\delta -0.2935e^{-z}z\delta +0.0733e^{-3z}\alpha_{2}\\ & -0.5871e^{-2z}\alpha_{2}+0.5137e^{-z}\alpha_{2}-0.5871e^{-z}z\alpha_{2}\\ & +0.2201e^{-3z}\delta \alpha_{2}-1.1742e^{-2z}\delta \alpha_{2}+0.9540e^{-z}\delta \alpha_{2}\\ & -0.8806e^{-z}z\delta \alpha_{2}+0.1467e^{-3z}\delta^{2}\alpha_{2}-0.5871e^{-2z}\delta^{2}\alpha_{2}\\ & +0.4403e^{-z}\delta^{2}\alpha_{2}-0.2935e^{-z}z\delta^{2}\alpha_{2}-\frac{0.1957e^{-2z}\gamma_{2}}{\mathrm{Sc}}\\ & +\frac{0.1957e^{-z}\gamma_{2}}{\mathrm{Sc}}. \end{array}$$

## 4. Analysis and Discussion

**Figure 2:**
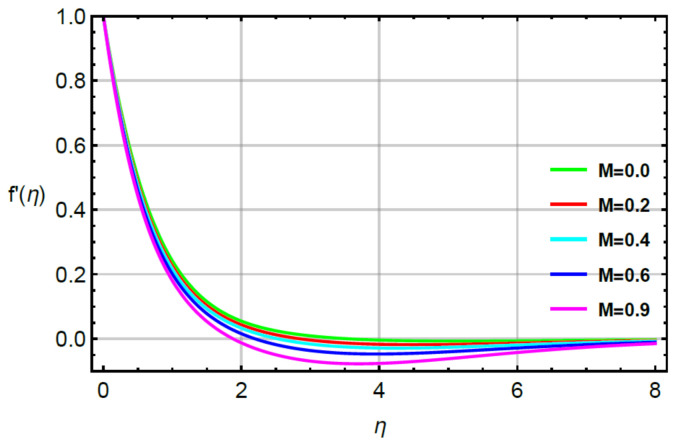
Behavior of $f^{\prime }\left(\eta \right)$ for M when $R=0.1,Sc=0.6,Pr=1.1,\;\;\alpha_{1}=0.2=\alpha_{2},\gamma_{1}=0.3=\gamma_{2},\delta =0.8.$

**Figure 3:**
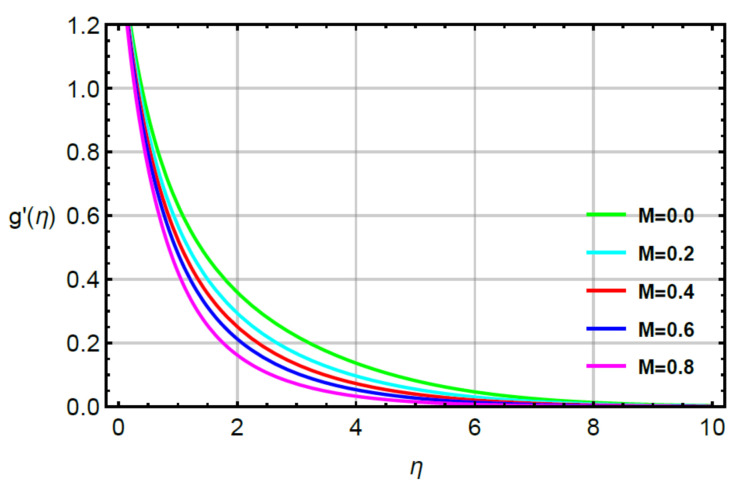
Behavior of $g^{\prime }\left(\eta \right)$ for M when $R=0.1,Sc=0.6,Pr=1.1,\;\;\alpha_{1}=0.2=\alpha_{2},\;\;\gamma_{1}=0.3=\gamma_{2},\delta =0.8.$

**Figure 4:**
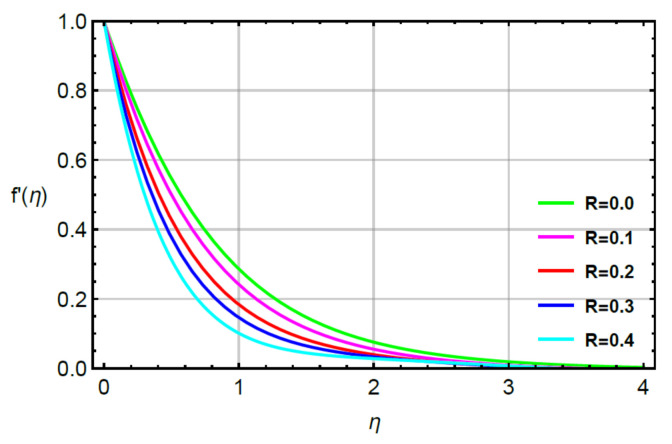
Behavior of $f^{\prime }\left(\eta \right)$ for R when $Sc=0.6,Pr=1.1,\;\;\alpha_{1}=0.2=\alpha_{2},\gamma_{1}=0.3=\gamma_{2},M=0.1,\delta =0.8.$

**Figure 5:**
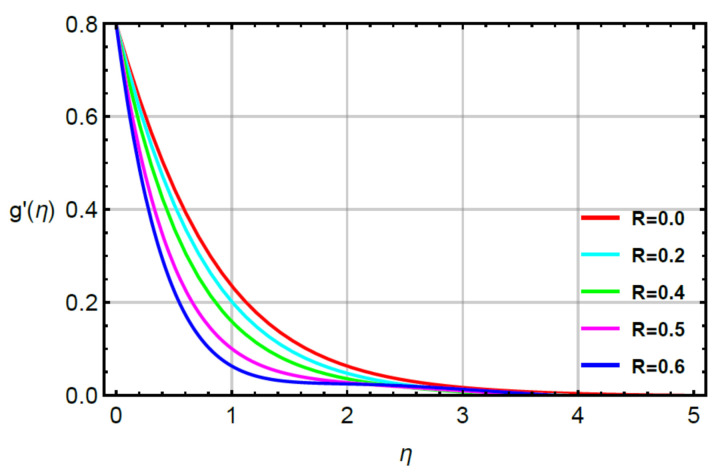
Behavior of $g^{\prime }\left(\eta \right)$ for R when $Sc=0.6,Pr=1.1,\;\;\alpha_{1}=0.2=\alpha_{2},\gamma_{1}=0.3=\gamma_{2},M=0.1,\delta =0.8.$

**Figure 6:**
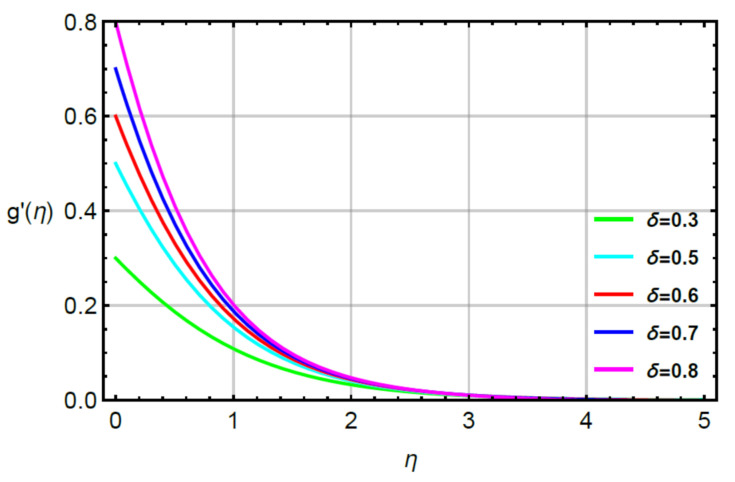
Behavior of $g^{\prime }\left(\eta \right)$ for δ when $R=0.1,Sc=0.6,Pr=1.1,\;\;\alpha_{1}=0.2=\alpha_{2},\gamma_{1}=0.3=\gamma_{2},M=0.1.$

The assessments of vital applications of thermal energy and mass transport (using second grade liquid) in industrial and engineering areas are addressed, including various features. In this current problem, non-Fourier’s theory is investigated in energy and mass transport equations along with the concept of variable properties (mass diffusion and thermal conductivity). The motion in nanoparticles is induced because of movement of a melting 3D-surface. The simulations of temperature, diffusion of mass and flow behavior are captured in the form of graphs and tables via an analytical approach. The error analysis is presented with the help of Table 1. The graphical discussions are addressed below:

**Assessments of flow phenomena via physical parameters:** the distribution of flow phenomena is analyzed with respect to magnetic number (M), second grade fluid number (R) and velocity stretching ratio number (δ). In this current problem, M is considered as variable magnetic number for measurement of flow behavior in both directions (vertical and horizontal). The variation strength of a magnetic field is considered during the flow of particles at the surface of the melting sheet. This effect is analyzed by Figure 2 and Figure 3. The decreasing character in the motion of particles is noticed via enhancement strength regarding magnetic field. The declination in flow is due to a negative force called Lorentz force appeared in momentum equations. The retardation forces play a reducing role in motion of fluid particles. Therefore, a magnetic number is used to adjust (MBL) momentum boundary layer thickness. It is noticed that the last terms of dimensionless momentum equations represent negative Lorentz forces. These negative Lorentz forces generate hindrance during the flow of fluid particles. The reduction is investigated in velocity profiles for M=0.0, 0.2, 0.4, 0.6, 0.8. Therefore, a local minimum trend is noticed for M=0.0, 0.2, 0.4, 0.6, 0.8. The parameter related to R is known as a second grade number whereas the character of R is simulated on the flow behavior. In this case, the reduction in flow mechanism is conducted through the numerical values of R and this graphical impact is considered in Figure 4 and Figure 5. It is noticed that the appearance of R is modeled due to the appearance of second grade fluid in the current model. Meanwhile, flow in the horizontal and vertical directions is reduced, taking higher values of R. Moreover, flow in the absence of R is higher as compared with flow situation in the presence of R. Simply, it is included that flow for the case Newtonian liquid becomes higher than as compared flow for the case of non-Newtonian liquid. Figure 6 provides the role of δ on distribution of velocities. The enhancement in velocity profiles is conducted using higher values of δ while the parameter related to δ is addressed as velocity ration parameter. From these figures, fluid speed is enhanced via more stretching of melting surface. The large stretching of the surface is the reason for the more enhancements in fluid motion. Hence, δ is favorable to achieve the enhancement in motion of the fluid particles.

**Figure 7:**
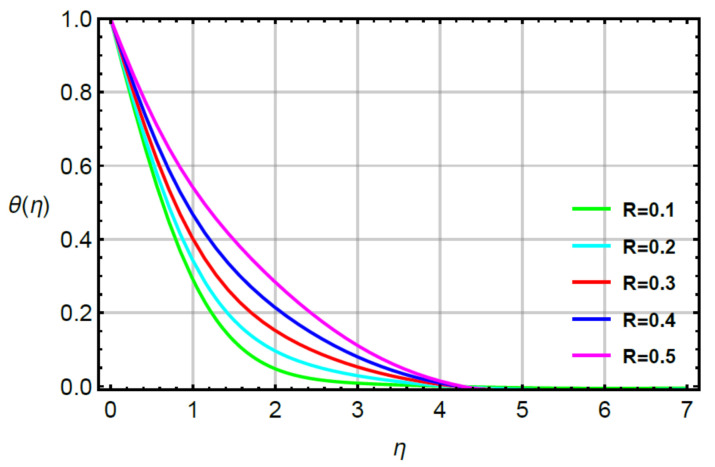
Behavior of $\theta \left(\eta \right)$ for R when $Sc=0.6,Pr=1.1,\alpha_{1}=0.2=\alpha_{2},\;\;\gamma_{1}=0.3=\gamma_{2},M=0.1,\delta =0.8.$

**Figure 8:**
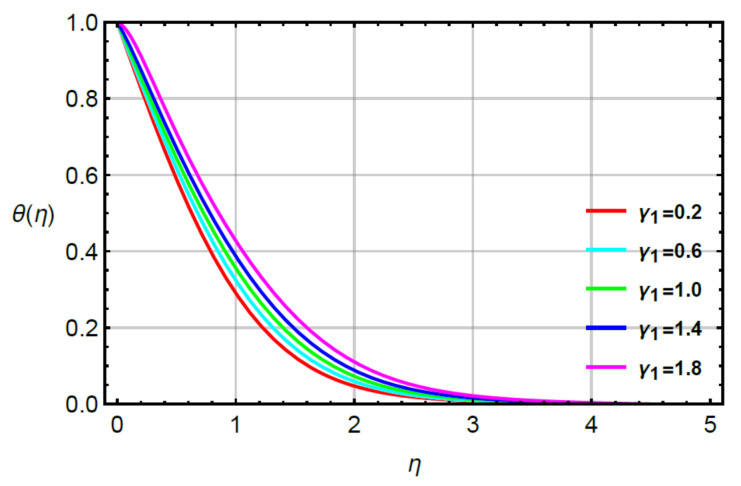
Behavior of $\theta \left(\eta \right)$ for $\gamma_{1}$ when $R=0.1,Sc=0.6,Pr=1.1,\alpha_{1}=0.2=\alpha_{2},\gamma_{2}=0.3,M=0.1,\delta =0.8.$

**Figure 9:**
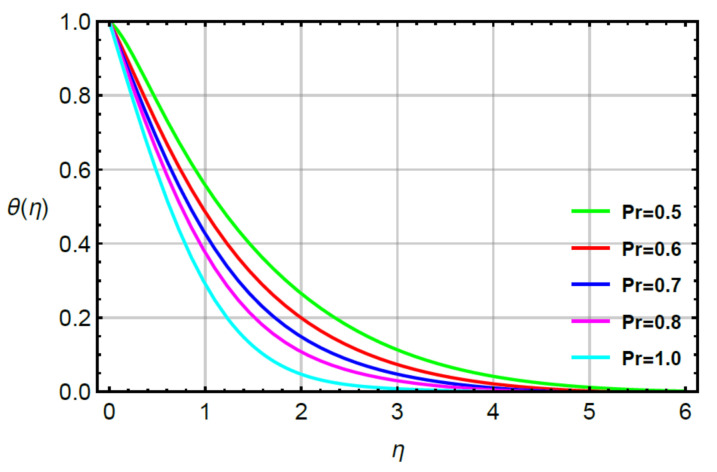
Behavior of $\theta \left(\eta \right)$ for Pr when $R=0.1,Sc=0.6,\;\;\alpha_{1}=0.2=\alpha_{2},\;\;\gamma_{2}=0.3,M=0.1,\delta =0.8.$

**Figure 10:**
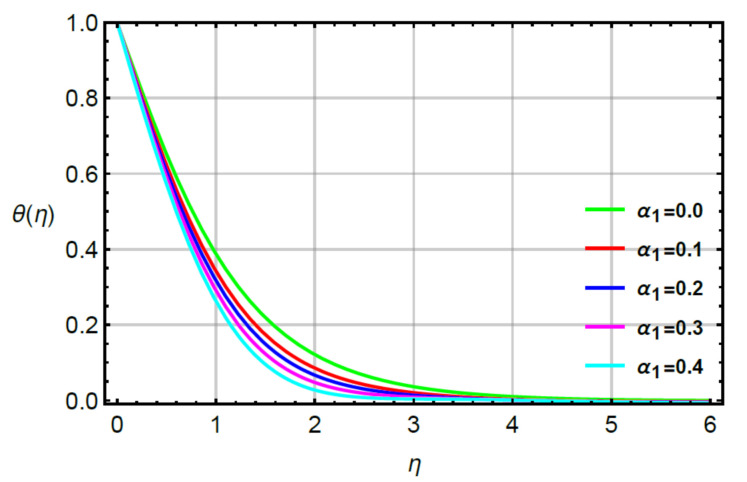
Behavior of $\theta \left(\eta \right)$ for $\alpha_{1}$ when $R=0.1,Sc=0.6,\alpha_{2}=0.2,\gamma_{2}=0.3,M=0.1,\delta =0.8.$

**Assessments of heat energy via physical parameters:** the characterization of the thermal energy mechanism against the variation in $Pr,\;\alpha_{1},\;\Upsilon_{1}$ and R is conducted in Figure 7, Figure 8, Figure 9 and Figure 10. In Figure 7, the role of second grade fluid number is considered. The mechanism of thermal energy is reduced using higher values of R. The thickness of thermal boundary is also decreased. The characterization of heat energy against the distribution of very small number ($\Upsilon_{1}$) based on thermal conductivity is measured by Figure 8. The production of heat energy becomes higher via higher values of $\Upsilon_{1}.$ Hence, $\Upsilon_{1}$ plays an essential role in the enhancement of maximum production of energy. The performance of Prandtl number versus thermal energy is visualized by Figure 9. The temperature profile is noticed as reducing the role versus the variation of Pr. This reducing role happens because of the definition of the Prandtl number. According to the definition of Pr, MBL is enhanced while the thickness of TBL is increased. Meanwhile, the production of heat energy is reduced with respect to large values of Pr. Figure 10 illustrates the trend of $\alpha_{1}$ versus the temperature profile and $\alpha_{1}$ is denoted as a relation time parameter. It is investigated how $\alpha_{1}$ has appeared due to Cattaneo heat flux theory in the energy equation. The thermal energy is reduced due to attendance of non-Fourier’s concept whereas thermal energy for the case of Fourier’s law is higher than thermal energy for the case of the non-Fourier’s concept.

**Figure 11:**
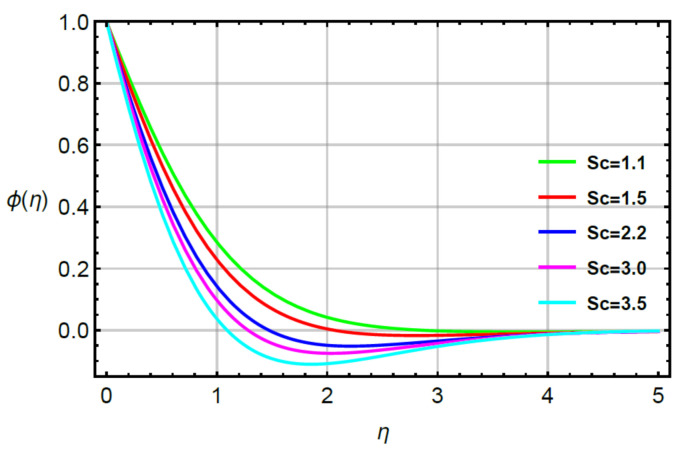
Behavior of $\phi \left(\eta \right)$ for Sc when $R=0.1,\;\;\alpha_{1}=0.2=\alpha_{2},\;\;\gamma_{2}=0.3,\;\;M=0.1,\;\;\delta =0.8$.

**Figure 12:**
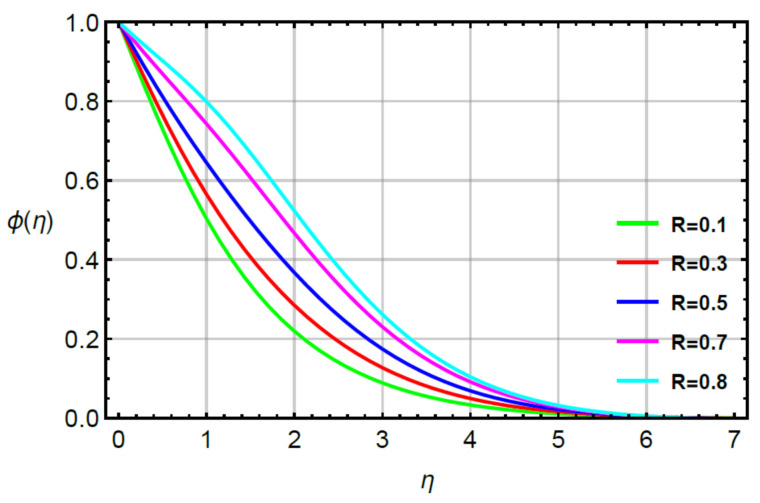
Behavior of $\phi \left(\eta \right)$ for R when $Sc=0.6,\alpha_{1}=0.2=\alpha_{2},\gamma_{2}=0.3,M=0.1,\delta =0.8.$

**Figure 13:**
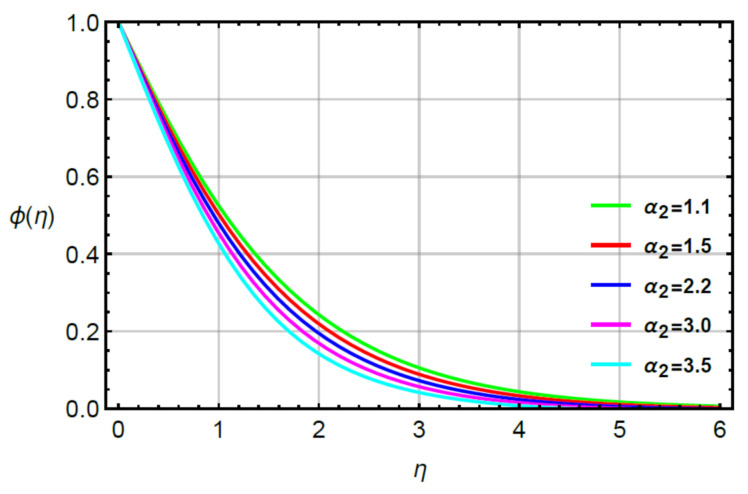
Behavior of $\phi \left(\eta \right)$ for $\alpha_{2}$ when $R=0.1,Sc=0.6,\alpha_{1}=0.2,\;\;\gamma_{2}=0.3,M=0.1,\delta =0.8.$

**Figure 14:**
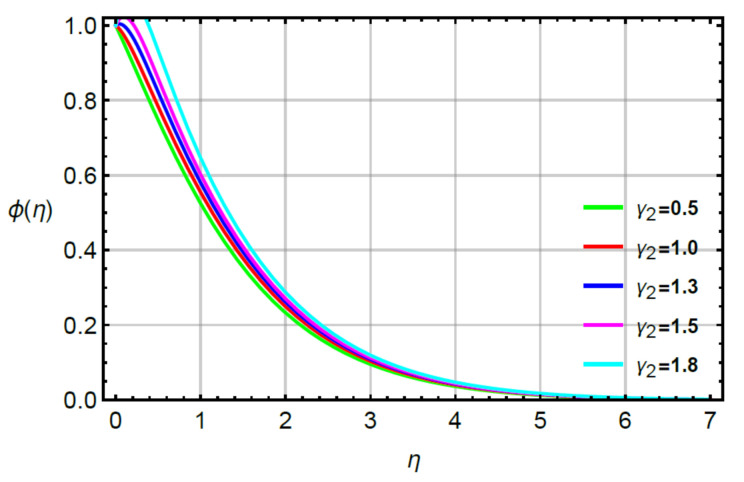
Behavior of $\phi \left(\eta \right)$ for $\gamma_{2}$ when $R=0.1,Sc=0.6,\alpha_{1}=0.2=\alpha_{2},M=0.1,\delta =0.8.$

**Assessments of mass diffusion via physical parameters:** the distribution in transport of mass diffusion is measured against the variation in $Sc,\;\alpha_{2}$ and $\gamma_{2}$ via Figure 11, Figure 12, Figure 13 and Figure 14. The mechanism related to mass diffusion versus Sc called the Schmidt number is conducted by Figure 11. From this figure, declination is measured in view of the transport of mass using large values of the Schmidt number. The transport of mass becomes slow due to the concept of Sc. According to this definition, the diffusion of mass (ratio between momentum and mass diffusivities) has an inverse relation with respect to Sc. Hence, the solute slows down considering enlargement in Sc. Due to this depreciation occurs in the concentration field. Moreover, the combined enlargement in the Schmidt number and solutal relaxation time lessen the concentration profile. The decreasing graph of the concentration profile is observed versus the values of Sc. This decreasing trend and local minimum in solute particles is occurred due to reduction of mass diffusivity. Physical, large values of Sc reduce mass diffusivity and this reduction in mass diffusivity is a reason for the local minimum in solute particles. The better performance of solute is the investigated against variation in R considering by Figure 12. The range of R is 0.1≤R≥0.8 is used to obtain maximum production of solute. Figure 13 simulates the variation in mass transport with respect to values of $\alpha_{2}.$ The decrement in solute is verified considering large values of $\alpha_{2}.$ This physical parameter has the ability to restore a state of equilibrium resulting in the solute becoming slow. Figure 14 captures the role of $\gamma_{2}$ versus the diffusion of mass. In this figure, diffusion of mass becomes fast using large values of $\gamma_{2}.$

**Assessments of Nusselt number, divergent velocity and Sherwood number via physical parameters:** the characterization of surface force, temperature gradient and rate of solute is analyzed considering large values of second grade fluid, stretching ration and time relaxation numbers. These simulations are captured by Table 2 and Table 3. From Table 2, it can be noted that an enhancement is addressed in the case of positive values of second grade fluid and time relaxation numbers. Table 2 presents the comparative analysis of current model. The remarkable simulations are verified with published work [7]. Table 4 illustrates the impact of time relaxation numbers against the diffusion of mass. The reparable increasing role of the Sherwood number is measured against positive values of the time relaxation number.

## 5. Conclusions and Key Findings of the Investigation Performed

The physical occurrence of solute, heat energy and flow phenomena in a second grade liquid were visualized passing a 3D melting moving surface. The theory of non-Fourier’s law was imposed in the current flow model inserting variable properties. The current complex model was simulated with the help of an analytical scheme. The main consequences of the current problem are addressed below:

- The improvement in motion of fluid particles was captured via large values of second grade fluid and stretching ratio numbers while a decrement in flow behavior was conducted via enlargement in magnetic number;
- The mechanism of heat energy became maximum using higher values of second grade fluid number but an opposite trend was captured via large values of time relaxation, Prandtl and very small numbers;
- The solute became fast considering large values of second grade fluid, time relaxation and very small numbers. The reduction in solute became slow against variation in Schmidt number;
- An incline in rate of solute and gradient temperature was addressed against higher values of time relaxation numbers;
- The surface force was enhanced near the wall of the hot surface via large values of second grade liquid and flow stretching parameters.

## Figures and Tables

**Table 1:** Computation of averaged squared residuals errors of velocity, temperature, and concentration solution.

$\left(B\right)$	$\delta_{B}^{f}$	$\delta_{B}^{g}$	$\delta_{B}^{\theta }$	$\delta_{B}^{\phi }$
1	0.0003	0.0001	0.0008	0.0130
4	$6.612\times 10^{-6}$	$3.231\times 10^{-6}$	$5.461\times 10^{-6}$	0.00017
8	$3.653\times 10^{-7}$	$1.934\times 10^{-7}$	$1.787\times 10^{-7}$	$8.412\times 10^{-6}$
12	$3.068\times 10^{-8}$	$1.661\times 10^{-8}$	$8.074\times 10^{-9}$	$7.117\times 10^{-7}$
16	$4.169\times 10^{-9}$	$2.265\times 10^{-9}$	$1.063\times 10^{-9}$	$1.076\times 10^{-7}$
20	$5.760\times 10^{-10}$	$3.108\times 10^{-10}$	$1.522\times 10^{-10}$	$1.850\times 10^{-8}$

**Table 2:** Comparative analysis for dimensionless stress against R and γ by fixing the other parameters.

R	γ	$-{\left(R_{e_{xy}}\right)}^{\frac{1}{2}}C_{Fx}^{*}$ [7]	Present	$-{\left(R_{e_{xy}}\right)}^{\frac{1}{2}}C_{Fx}^{*}$ [7]	Present
0.0	0.1	1.0203	1.0209	0.0669	0.0668
0.15	-	1.6510	1.6519	0.0785	0.0784
0.2	-	1.8970	1.8975	0.0806	0.0803
0.1	0.0	1.3703	1.3707	0.0000	0.0000
-	0.2	1.4798	1.4798	0.1762	0.1769
-	0.5	1.6510	1.6516	0.6317	0.6312

**Table 3:** Comparative investigation for heat transfer rate against $\alpha_{1}$.

$\alpha_{1}$	$-{\left(R_{e_{xy}}\right)}^{\frac{1}{2}}\theta^{\prime }\left(0\right)$ [7]	Present Results
0.0	0.6051	0.6059
0.2	0.6258	0.6256
0.4	0.6483	0.6489
0.6	0.6727	0.6729

**Table 4:** Comparative investigation for mass transfer rate against $\alpha_{2}$.

$\alpha_{2}$	$-{\left(R_{e_{xy}}\right)}^{\frac{1}{2}}\phi^{\prime }\left(0\right)$ [7]	Present Results
0.0	0.3668	0.3669
0.2	0.3764	0.3760
0.4	0.3864	0.3862
0.6	0.3973	0.3978

## Data Availability

The data used to support this study are included in the manuscript.

## Nomenclature

**Symbols**	**Used for**	**Symbols**	**Used for**
$“U_{W}=ax”$ and $“V_{W}=by”$	Velocity at wall	x, y, z	Space coordinates
u, v, w	Dimensional velocity	ϑ	Kinematic viscosity
$B_{a}^{2}\left(x,y\right)$	Non-uniform magnetic field	σ	Electrical conductivity
ρ	Fluid density	δ	stretching ratio number
$-{\left(R_{e_{xy}}\right)}^{\frac{1}{2}}\theta^{\prime }\left(0\right)$	Heat transfer rate	$-{\left(R_{e_{xy}}\right)}^{\frac{1}{2}}\phi^{\prime }\left(0\right)$	Mass transportation rate
TBL	Thermal boundary layer	MBL	Momentum boundary layer
$\begin{array}{c} L_{f}^{*}=\frac{D^{3}}{D\eta^{3}}-\frac{D}{D\eta },\;L_{g}^{*}=\frac{D^{3}}{D\eta^{3}}-\frac{D}{D\eta },\;\\ L_{t}^{*}=\frac{D^{2}}{D\eta^{2}}-1,\;L_{c}^{*}=\frac{D^{2}}{D\eta^{2}}-1,\; \end{array}$	Linear operators	$\begin{array}{l} f_{q}\left(\eta \right)=1-e^{-\eta },\;g_{q}=\gamma \left[1-e^{-\eta }\right],\;\\ \theta_{q}\left(\eta \right)=e^{-\eta },\;\phi_{q}\left(\eta \right)=e^{-\eta }, \end{array}$	Initial guesses
$\phi \left(\eta \right)$	Dimensionless concentration	$\theta \left(\eta \right)$	Dimensionless temperature
η	Dimensionless independent variable	Pr	Prandtl number
$\alpha_{b}$	Concentration relaxation time	$\alpha_{a}$	Temperature relaxation time
Sc	Schmidt number	$f^{\prime }\left[\eta \right],g^{\prime }\left[\eta \right],\;f\left[\eta \right],\;g\left[\eta \right]$	Dimensionless velocity
R	Fluid parameter	M	Magnetic parameter
